# Prosecution and Conviction of “an American Physician” for Illegally Practising as an Apothecary in England

**Published:** 1846-10

**Authors:** 


*Prosecution and conviction of "an American Physician" for illegally practising as an Apothecary in England.*

Bristol Summer Assizes, 17th August, 1846. The Master Wardens and Society of Apothecaries of London *v.* Frank Bargeer Wall. Before Mr. Justice Earle and a Special Jury.

Mr. Barstow having opened the pleadings,

Mr. Cockburn said,—This is an action brought by the Society of Apothecaries, under the 55th of George III, chapter 194, which makes it penal to practise within this realm as an apothecary, without having obtained a certificate of qualification from the Society of Apothecaries. This is a matter of very considerable importance to the life and health of the subjects of this country. You can understand, that if an unlimited license were allowed to all persons, whether qualified or not, to practice as medical men, the consequences might be most fatal. And in this, as, indeed, in every other civilized country, a system, under some shape or modification, of medical police and regulation has been thought necessary for the protection of the public. Accordingly, this Act was passed, new modelling and giving new powers to the Society of Apothecaries, and intrusting them with the performance of the important duties which attach to them under its provisions. (The learned council here read the sections of the Act which appoint a Court of Examiners, and render it penal for any person to practise as an apothecary who has not undergone an examination and obtained a certificate of qualification.) If, therefore, any person shall practise as an apothecary, except he has received such certificate, he shall forfeit 20*l*., to be recovered by action at the suit of the Society, whose bounden duty it is, if they discover any person so practising,—whose sacred duty it is, because the safety of the public is involved in its discharge,—to enforce the provisions of the Act against persons who choose to infringe upon its provisions. This is not, therefore, a common suit at the instance of an informer. The Society have no private interests to serve, no private motives to gratify, but simply a public duty to discharge in instituting the present proceedings. The defendant in this case has, as I can show you in various instances, infringed the provision of the Act in this town. He practised as an apothecary in the cases which my learned friend brought under your notice in opening to you this case. To all intents and purposes he is carrying on business as if he had been examined by, and received a certificate from, the Apothecaries' Society, although he never submitted himself to the examination, and is in possession of no certificate which justifies him in so practising. These circumstances having been brought under the notice of the Society, they felt that they had but the one straight-forward duty to fulfil, of instituting these proceedings. I am not instructed to say a word against the gentleman's competency; he may be qualified, or he may not. I know nothing on the subject. If he be qualified, he has nothing to do but to submit himself to the examination, which, if he has the necessary qualification from education, he may readily undergo; and if he has not that qualification, he certainly ought to be prohibited from practising. Therefore he cannot in any way complain of these proceedings. I believe I shall have no difficulty whatever in substantiating the facts against the defendant. I do not know whether any difficulty will be attempted to be raised as to what constitutes the practice of an apothecary. For myself, I am provided with numerous authorities to show that the class of cases which he has affected to treat, (not being surgical, but medical cases—diseases of the brain, the lungs, the heart, the stomach, and so on) fall within the province of the apothecary. There is not the slightest doubt, from the decision of the Jndges, and the authority of the courts, that all these classes of cases come within the proper definition of practising as an apothecary.

Mr. Stone, (for the defendant) here interposed, and said, I take it for granted that you do not seek to recover more than one penalty in this case?

Mr. Cockburn.--I do not know. What is it that you propose to say?

Mr. Stone.—That we cannot resist this action, and that the Apothecaries' Society have done nothing more than their duty in instituting these proceedings. Unfortunately, this gentleman was not aware of the law which rendered it necessary for him to take out his certificate. He is a gentleman highly competent to practise; he has been practising as a physician in America; he served a regular apprenticeship in this city; he came back in November, 1844, and he certainly has been practising as an apothecary, since that, at the times you have stated. I take it for granted, that the only object the Apothecaries' Society can have is to compel him to submit himself to the ordinary examination, and to take out his certificate.

Mr. Cockburn.—Certainly.

Mr. Stone.—All I can say is, that he is a gentleman of high medical and surgical attainments. He has erred in mistake as to the law.

Mr. Cockburn.—Let the gentleman be all that my friend describes, that is the more abundant reason why he should conform to the law. If my friend says that he does not wish to resist this action, I am satisfied to take one penalty, with this condition, that as there are costs on the other issues, the arrangement should be made upon payment of the general costs of the action.

Mr. Stone.—Of course.

The jury, under the direction of the Judge, then returned a verdict for all the penalties in the declaration, amounting to £160, on the understanding that execution should issue only for one penalty and the general costs of the action.—*Lancet.*

